# Internal preference mapping of milk–fruit beverages: Influence of color and appearance on its acceptability

**DOI:** 10.1002/fsn3.494

**Published:** 2017-10-21

**Authors:** Rocío Fernández‐Vázquez, Carla M. Stinco, Dolores Hernanz Vila, Francisco J. Heredia, Carolina Chaya, Isabel M. Vicario

**Affiliations:** ^1^ Food Colour & Quality Laboratory Department of Nutrition & Food Science Facultad de Farmacia Universidad de Sevilla Sevilla Spain; ^2^ Department of Analytical Chemistry Facultad de Farmacia Sevilla Spain; ^3^ Department of Agricultural Economics Statistics and Business Management Universidad Politécnica de Madrid Spain

**Keywords:** acceptance, color, consumers study, milk–fruit beverage

## Abstract

The individual preferences of 100 consumers between 20 and 30 years old for the color of 16 milk–fruit juice beverages (MFJB) were investigated by preference mapping technique. Consumers were asked to evaluate, just by looking at the samples, how much they liked them (from “Extremely dislike” to “Extremely like”). The color of the samples was analyzed by two different instrumental techniques. Results obtained from the instrumental color measurement showed the wide diversity in hues of the beverages available in the market, and correlations between techniques proved that both of them were appropriate to analyze color.

Results showed that participants preferred samples with orangish appearance instead of those with a whiter look. Anyway, punctuations given by the consumers suggest that generally, color of these products is not highly evaluated by consumers, as the best mean punctuation was 6.6.

## 1 INTRODUCTION

Beverages made from milk and fruit juice (MFJB) have proliferated in the European market during the last years in response to a growing demand for natural products that are perceived as healthier by consumers. These beverages are considered the most widely consumed functional foods (Pszczola, 2005); however, there are little data related to quality, safety, or acceptability of these products (Sampedro, Geveke, Fan, Rodrigo, & Zhang, 2009).

From a nutritional point of view, they do not replace or are not equivalent to a glass of milk or a portion of fruit. Thus, in its composition, fruit contents range from 7% to 41% and they usually come from different concentrated fruits; milk means up to 30%, and they contain also vitamins, fiber, and sugars.

Instrumental color measurements are simple, accurate, versatile, and objective. To characterize color, there are different instruments, such as colorimeters, spectrophotometers, and spectroradiometers, and independently of the instrument used, it is very important to define measurements conditions correctly. CIE (International Commission on Illumination) recommends observers (CIE, 1991a), illuminants (CIE, 1991b), and color spaces (CIE, 1978), in order to normalize objective color measurement. It must be also taken into account sample position, system geometry, light source intensity, sample deepness, blank measurement, background, and surrounding (Meléndez‐Martínez, Vicario, & Heredia, 2006; Stinco et al., 2012). Digital image analysis (DIA) is being used increasingly for instrumental food color specification although more traditional techniques like spectroradiometry (SPE) will also provide an appropriate characterization of color in beverages (Fernández‐Vázquez, Stinco, Hernanz, Heredia, & Vicario, 2013, 2014; Stinco, Fernámdez‐Vázquez, Heredia, Meléndez‐Martínez, & Vicario, 2014).

Furthermore, color is one of the most important attributes related to quality, affecting choice of purchase (Baker & Günter, 2004; Calvo, Salvador, & Fiszman, 2001). For the consumers, color is, alongside freshness, one of the main criteria for food selection, and a key factor in sensory acceptance (Fernández‐Vázquez, Stinco, Melendez‐Martinez, Heredia, & Vicario, 2011; Quitao‐Teixeira, Aguiló‐Aguayo, Ramos, & Martín‐Belloso, 2008). Traditionally, this kind of products has been often consumed in their single‐use packages, meaning that consumers could not perceive the color, which might be the reason why industries have not put too much attention to this characteristic of the beverages. However, currently, due to the increasing in the consumption, many of the companies are changing the packages of the beverages to some with more volume, which means consumers will drink them in glasses, and therefore, they will perceive and evaluate their color.

Despite the relevance of milk–fruit beverages MFJB in the market, up to now there is a lack of information about the color preferences for these products and its consumer acceptance. Thus, the aims of this study were (1) to characterize the color of MFJB beverages using different techniques, (2) to study the consumers' acceptance of their color, and (3) to identify and characterize different consumers segments in terms of color acceptance by means of internal preference mapping.

## 2 MATERIALS AND METHODS

### 2.1 Samples

Sixteen commercially available MFJB formulated with milk or dairy products and fruit juice were purchased in different supermarkets in Spain, and their compositions are shown in Table 1. Three of the samples are sold at refrigeration temperature (4 ± 2°C) as they were pasteurized, while the other 13 samples are sold at room temperature (20 ± 2°C) since they were submitted to a UHT process.

**Table 1:** Composition of the commercial beverages analyzed

Samples	Main ingredients	Colorant	Fiber	Vitamins	Jelling agents	Sugar added	Other
S1^a^	Skimmed milk, concentrate orange 5%	B‐Carotene	1% inulin	–	–	7.6% sugar	Corn dextrose, ascorbic acid, citric acid, aromas, milk proteins, guar gum, lactobacillus acidophilus, bifidobacterium, plant sterols, and sterols esters
S2	Water, juice from concentrate: pineapple, carob, apple and grape, orange and lemon (12%), skimmed milk (4.5%)	B‐Carotene	–	–	Pectin	Sugar, sucralose, acesulfame‐K	Milk protein, citric acid, aromas, ascorbic acid
S3^a^	Partially skimmed milk, orange juice from concentrate (5%)	B‐Carotene	1% inulin	–	–	7.6% sugar	Corn dextrose, sodium citrates, ascorbic acid, guar gum, milk proteins, lactobacillus acidophilus, milk enzymes, bifidobacterium
S4^a^	Skimmed milk, 6% concentrate (orange, pineapple, mango)	B‐Carotene	Gum Arabic	–	–	Sugar	Bifidobacterium, milk enzymes, aromas
S5	Water, fruit juice: orange, apple, pineapple and lemon (15%), skimmed milk (10%)	–	–	–	Pectin	Sugar and sucralose	Citric acid, ascorbic acid, aromas
S6	Water, fruit juice: pineapple, orange, apple and lemon (15%), skimmed milk (10%)	–	–	–	Pectin	Sugar, acesulfame‐K, neohesperidin DC	Citric acid, ascorbic acid, aromas
S7	Water, fruit juice: orange (25%), apple (18%), pineapple (5%), lemon (1%), skimmed milk (10%)	–	Fiber	A, C, E	Pectin	Glucose and fructose (syrup), sugar	Citric acid, aromas
S8	Water, fruit, and vegetable juice: orange, grape, pineapple, carrot, peach, and passion fruit (25%), skimmed milk 8%	B‐Carotene	–	–	Pectin	Sucralose, acesulfame‐K	Citric acid, ascorbic acid, aromas
S9	Water, fruit, and vegetable concentrate juice: orange (8%), carrot (5%), lemon (2%), pineapple (2%), passion fruit (2%), skimmed milk (10%), puree peach (4%)	B‐Carotene	Dietary fibers	C, E	Pectin, xanthan gum	Fructose (syrup), sucralose	Citric acid, aromas
S10	Water, skimmed milk (10%), Concentrate: pineapple, mango (7%)	B‐Carotene	–	A, C, E	Pectin	Sugar, sucralose	Citric acid, aromas
S11	Water, fruit, and vegetable juices: orange, carrot, peach (15%), skimmed milk (10%)	B‐Carotene	–	–	Pectin	Saccharose	Citric acid, ascorbic acid, aromas
S12	Water, concentrate fruit juice: orange, puree peach, carrot (18%), skimmed milk (10%)	B‐Carotene	–	A, C, E	Pectin	Sucralose	Citric acid, aromas
S13	Water, fruit, and vegetable juice: orange, carrot, pineapple, passion fruit, mango, guava, papaya, and apricot (25%), skimmed milk (10%)	–	Fiber	A, C, E	Pectin	Sugar	Water, citric acid, aromas
S14	Water, juice from concentrate: orange, pineapple, apple, papaya, carrot, mango, guava (20%), skimmed milk (10%)	B‐Carotene	–	C, E	Pectin	Sugar, glucose, and fructose (syrup), sucralose	Citric acid
S15	Water, juice from concentrate: orange, apple, and mango, skimmed milk (10%)	B‐Carotene	–	A, C, E	Pectin	Sucralose, acesulfame‐K	Citric acid, aromas
S16	Water, juice from concentrate: 8% orange, 1% mango, 1% pineapple, skimmed milk (10%)	B‐Carotene	–	E	Pectin	Sugar	Citric acid, sodium citrates

a Samples stored under refrigeration.

### 2.2 Color measurements

#### 2.2.1 Spectrophotometry (SPE)

The color of the beverages was measured in a spectrophotometer CM5 (Konica Minolta Sensing Americas, Inc., NY). Each sample was contained in 75‐ml capacity transparent plastic bottles. The color parameters of the uniform color space CIELAB L*; a*; and b* were obtained directly from the apparatus. Color data obtained were averages of three measurements.

#### 2.2.2 Digital image analysis (DIA)

The DigiEye imaging system was used to capture the digital images (Luo, CUI, & LI, 2001). The latter system includes a calibrated digital camera with 10.2‐megapixel Nikon D80 (Nikon Corporation, Tokyo, Japan) and an objective Nikkor 35‐mm f/2D (Nikon Corporation), a color sensor for display calibration, and an illumination box designed by VeriVide Ltd. (Leicester, UK).

The samples were placed in 75‐ml capacity transparent plastic bottles, illuminated by a diffuse D65 simulator, and measured against a gray surround (L* = 50) and white background.

To calculate the CIELAB coordinates from RGB color space, the DigiFood software was used (Heredia, González‐Miret, Álvarez, & Ramírez, 2006).

From the CIELAB uniform color space, the psychophysical parameters chroma (C*ab) and hue (*h*_ab_) are defined as follows:

$$C_{\mathrm{ab}}^{*}=\sqrt{{\left(a^{*}\right)}^{2}+{\left(b^{*}\right)}^{2}},\;h_{\mathrm{ab}}=\text{arctan}\left(b^{*}/a^{*}\right)$$

Chroma ($C^{{*}_{\mathrm{ab}}}$) is used to determine the degree of difference of a hue in comparison with a gray color with the same lightness and is considered the quantitative attribute of colorfulness. Hue (*h*_ab_) is the attribute according to which colors are usually defined as reddish, greenish, etc. and is used to define the difference of a color with reference to a gray color with the same lightness. This attribute is related to the differences in reflectance at different wavelengths and is considered the qualitative attribute of color.

### 2.3 Consumer study

One hundred Spanish consumers were recruited from staff and students at the University of Sevilla. Information regarding demographics and consumption habits was collected via a questionnaire prior to the sensory assessment of the samples. All the consumers were 20–30 years old (30% males and 70% females), which is especially interesting as it is the group of population which are potential consumers of these products.

The test was carried out in designed individual sensory booths, under Northern Hemisphere lighting conditions. Samples (75 ml) were presented monadically in the same bottles used for the other measurements, labeled with three digits random codes, in a randomized order.

Consumers were asked to evaluate how much they liked the appearance of the beverages (from “Dislike extremely” to “Like extremely”) using the 9‐point hedonic scale, just by looking at the samples. The rating decision was based only on the appearance, without further information.

### 2.4 Data analysis

The statistical analysis of instrumental color data was performed by one‐way analysis of variance (ANOVA), and statistically significant differences (*p < *0.05) were determined using the Tukey multiple comparison test. Correlation analysis was done between the colorimetric parameters measured by both instrumental techniques and between instrumental analysis and consumer study results.

Consumer data first underwent normality testing (Shapiro–Wilk test) and were subsequently analyzed using nonparametric tests (Kruskal–Wallis) to identify differences among samples. Then, these data were further examined using hierarchal cluster analysis, using Squared Euclidean Distances and Wards criterion, and internal preference mapping.

Independence between demographic variables and consumer clusters were analyzed by χ^2^ test.

All statistical analyses were performed using the program Statistica 8 for Windows (StatSoft, 2007) and XLStat (Version 2009.6.03, Addinsoft, USA).

## 3 RESULTS AND DISCUSSION

### 3.1 Instrumental color measurements

Table 2 shows the color coordinates of the beverages measured by the two techniques. Lightness (L*) values ranged from 61.75 to 90.91 in DIA and from 48.69 to 85.75 in SPE, thus samples seemed lighter when SPE was considered. Chroma (C*_ab_) values ranged from 21.55 to 57.52 in DIA but from 9.73 to 53.97 in SPE, showing again lower values, and wider range when SPE is considered. Finally, hue (*h*_ab_) values ranged from 52.58 to 87.91 in DIA and from 61.79 to 99.56 in SPE, which means that, when DIA is considered, samples seemed more reddish.

**Table 2:** Color coordinates of the beverages measured by the different techniques: spectroradiometer (SPE) and by digital image analysis (DIA)

Sample	Spectrophotometry			Digital image analysis		
	*L* a	*C* a	*h*_ab_	*L* a	*C* aab	*h*_ab_
S1^a^	85.75 ± 0.02^a^	21.53 ± 0.02^a^	75.01 ± 0.03^a^	90.91 ± 0.12^a^	22.05 ± 0.20^a^	77.90 ± 0.40^a^
S2	54.67 ± 0.09^b^	9.73 ± 0.04^b^	99.56 ± 0.08^b^	73.31 ± 0.13^b^	21.97 ± 0.20^a^	87.91 ± 0.18^b^
S3^a^	81.19 ± 0.01^c^	27.60 ± 0.03^c^	74.44 ± 0.03^c^	86.67 ± 0.07^c^	27.69 ± 0.11^b^	75.38 ± 0.09^c^
S4^a^	83.06 ± 0.02^d^	28.64 ± 0.08^c^	80.34 ± 0.03^d^	89.57 ± 0.09^d^	30.55 ± 0.12^c^	81.80 ± 0.22^d^
S5	55.64 ± 0.12^e^	21.69 ± 0.83^a^	86.48 ± 0.47^e^	72.31 ± 0.05^e^	30.66 ± 0.31^c^	78.59 ± 0.13^ae^
S6	49.03 ± 0.08^f^	14.63 ± 0.06^d^	94.14 ± 0.08^f^	70.40 ± 0.65^f^	28.80 ± 1.09^b^	86.83 ± 0.41^f^
S7	60.08 ± 0.07^g^	25.90 ± 0.09^e^	88.67 ± 0.14^g^	68.79 ± 0.11^g^	33.10 ± 0.14^d^	84.10 ± 0.06^g^
S8	50.86 ± 0.02^h^	51.84 ± 0.04^f^	62.08 ± 0.09^h^	61.75 ± 0.22^h^	50.48 ± 0.19^e^	54.24 ± 0.07^h^
S9	51.73 ± 0.18^i^	43.50 ± 0.10^g^	68.40 ± 0.03^i^	67.53 ± 0.02^ik^	51.40 ± 0.07^e^	62.47 ± 0.12^i^
S10	58.25 ± 0.02^j^	26.66 ± 0.12^ce^	83.12 ± 0.04^j^	76.15 ± 0.00^j^	42.93 ± 0.00^f^	79.21 ± 0.00^e^
S11	53.45 ± 0.14^k^	37.40 ± 0.15^h^	64.31 ± 0.05^k^	66.95 ± 0.00^i^	45.86 ± 0.00^g^	59.75 ± 0.00^j^
S12	53.04 ± 0.19 ^l^	36.36 ± 0.44^h^	61.95 ± 0.08^h^	67.76 ± 0.08^kl^	45.18 ± 0.08^g^	57.40 ± 0.15^k^
S13	48.69 ± 0.06^f^	43.91 ± 0.12^g^	61.79 ± 0.06^h^	62.01 ± 0.05^h^	51.54 ± 0.08^e^	52.59 ± 0.15 ^l^
S14	49.64 ± 0.01^m^	39.93 ± 0.11^i^	63.10 ± 0.06^l^	64.97 ± 0.22^m^	47.58 ± 0.22^h^	56.20 ± 0.14^m^
S15	57.28 ± 0.04^n^	53.97 ± 0.09^j^	81.28 ± 0.05^m^	65.71 ± 0.4^m^	57.87 ± 0.14^i^	81.69 ± 0.12^d^
S16	58.30 ± 0.00^j^	40.03 ± 0.04^i^	76.09 ± 0.03^n^	68.49 ± 0.03^gl^	57.52 ± 0.08^i^	70.63 ± 0.06^n^

^a‐o^ Different superscripts within columns for each parameter indicate statistically significant differences (*p *<* *0.05).

a Samples stored under refrigeration.

Zulueta, Esteve, and Frígola (2007) reported similar values, ranging from 53.05 to 80.62; 17.67 to 63.59; and 35.47 to 115.57; for L*, C*_ab_ and *h*_ab_, respectively, in different commercial MFJB analyzed with a Hunter Labscan II Colorimeter (Hunter Associates Laboratory, Inc., Reston, VA, USA).

These wide ranges in the values show the broad variety of color in the beverages available in the market. Thus, ANOVA (Table 2) showed that most of the samples were significantly different in all the colorimetric parameters.

The fruit/milk composition of the beverages is related to the color and thus to the colorimetric parameters and could explain color differences among samples. In this sense, samples stored under refrigeration (S1, S3, and S4), which were the samples with the highest values of lightness, had as main ingredient milk and its proportion of fruit was minor (5%–6% of concentrated fruit). This could explain the high values of lightness in those samples.

However, generally, beverages which contain mayor proportion of fruits with reddish‐yellowish colorations, such as orange, peach, mango, or carrot, presented hue values minor of 70°, while fruits with yellowish‐greenish colorations, such as apple, pineapple, or lemon, are associated with values of hue above 90°.

Regarding chrome, no significant relation between the composition and the colorimetric parameters could be argued.

#### 3.1.1 Relationship between instrumental techniques

**Figure 1:**
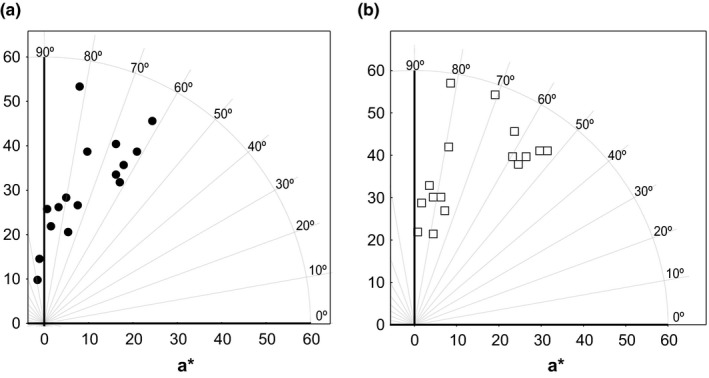
a*b* color diagram for juice–milk beverages measured by spectrophotometer (a) and digital image analysis (b)

Figure 1 shows the samples in the CIELAB space (Diagram a*b*) measured by SPE (Figure 1a) and DIA (Figure 1b). It can be observed that samples were situated in the same area of the CIELab diagram, independently of the technique used although with slight modifications in the positions. These slight variations observed in the color coordinates values measured by different techniques were also found in a previous study (Fernández‐Vázquez et al., 2011), where color of different orange juices was measured by DIA and SPE.

Instrumental measurements are considered an accurate and suitable method for evaluating color in food, as it has already been reported for wine and orange juice (Fernández‐Vázquez et al., 2011; Martínez, Melgosa, Pérez, Hita, & Negueruela, 2001). However, there might be differences among the measurements, depending on the technique used. They may be related mainly to differences in the thickness of the measured sample, other secondary factors such as illumination conditions, or the geometry of the system. In this sense, it is interesting to check if all the techniques are significantly correlated.

In this case, all the correlation coefficients were high (>0.85) and statistically significant (α = 0.05). Thus, it confirmed the relationship between both techniques in the measurements of the color of this kind of beverages.

### 3.2 Consumer study

Results from the demographic and consumption questionnaire are shown in Table 3. Results indicate that 85% of consumers considered these products as beneficial, and 70% consumed them quite often (39% did so two or more times per week). Participants who did not use to consume these beverages stated that they did not like them (63%), or they preferred other beverages, such as natural juices (20%).

**Table 3:** Demographic characteristics and consumption habits for each cluster and χ^2^ test results

	Frequency response (%)			*p*‐Value (χ^2^)^a^
	Cluster 1, *n *=* *19	Cluster 2, *n *=* *29	Cluster 3, *n *=* *52	
Gender				0.107
Male	11	38	33	
Female	89	62	67	
Consumption				0.202
Yes	63	83	67	
Frequency				0.300
<Once a week	42	28	29	
Once a week	–	14	14	
Twice a week	5	10	16	
>Twice a week	16	31	8	
No	37	17	35	
Reasons				–
High price	–	3	–	
It is not beneficial	–	–	1	
Don't like	32	3	22	
Other	5	11	12	
Opinion about if these products are beneficial				–
Yes	84	93	80	
No	16	7	20	

a When suitable, χ^2^ test was applied to test independence between clusters and demographic characteristics.

**Figure 2:**
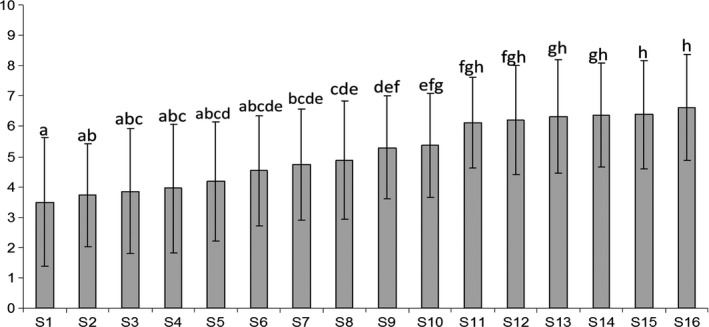
Mean values appearance scores given by consumers and significant differences identified by Kruskal–Wallis test

Figure 2 shows mean values given by consumers to the beverage appearances. It is worthy to highlight that the punctuations suggested that color of these products was not highly evaluated, as the best mean punctuation was 6.6. Anyway, generally, participants significantly preferred samples with orangish and more vivid appearance instead of those with more whitish look (Table 4). This could be due to the fact that consumers expected to find colorful beverages as they are supposed to be made with fruits, and when they observed beverages with whitish color, they may associate it with a lower fruit content. This apparently lower preference could also be due to the low mean consumption on the group. However, the consumers segment with highest rate of dislikers (Cluster 1, 32% dislikers, see Table 3) are the best discriminators between samples in terms of color appearance, reaching the highest mean scores in some samples.

**Table 4:** Mean acceptance scores and appearance of the samples (pictures taken with DigiEye System)

Samples	Acceptance scores	Samples	Acceptance scores	Samples	Acceptance scores	Samples	Acceptance scores
S1	3.5	S2	3.7	S3	3.9	S4	4.0
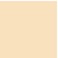		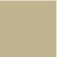		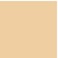		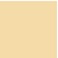	
S5	4.2	S6	4.5	S7	4.7	S8	4.9
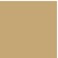		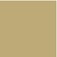		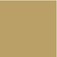		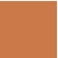	
S9	5.3	S10	5.4	S11	6.1	S12	6.2
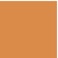		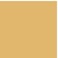		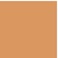		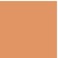	
S13	6.3	S14	6.4	S15	6.4	S16	6.6
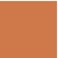		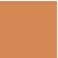		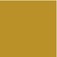		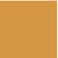	

Relation between consumer acceptance and color parameters was explored, and they showed that hue and chroma were significantly (*p* < 0.05) correlated with consumer acceptance, with the highest correlation coefficient for consumer acceptance and hue measured by DIA (*r* = 0.97).

#### 3.2.1 Cluster analysis

To find out if there were groups of consumers differing in their preferences for color, a segmentation of the panel group was done by Cluster analysis (Vigneau, Qannari, Punter, & Knoops, 2007). The three groups of consumers clearly identified, and their demography and consumption habits are shown in Table 3. Results from χ^2^ test showed that there were no significant differences in demographic characteristics or consumption frequency among clusters, indicating that these variables did not influence color acceptance patterns. Previous studies on consumer's color acceptance of different products like strawberry nectar from puree and orange juices showed similar results, where neither gender nor age or consumption habits had significant impact on color acceptance (Fernández‐Vázquez et al., 2011; Gossinger et al., 2009).

**Figure 3:**
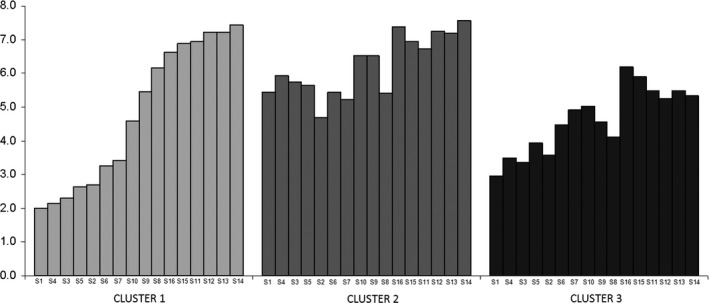
Mean appearance scores given by each of the clusters

Mean appearance scores given by the clusters are shown in Figure 3. The first cluster (19%) showed a clear preference for those samples with orangish appearance, giving average punctuations even higher than 7 (“Like moderately”) and also a deeply disliking for those with a whiter look, with punctuations lower than 2 (“Dislike very much”).

However, cluster 2 (29%) did not differentiate so much the samples. Thus, the worst valuated by this group of consumers was Sample 2 (average of 4.7) and the best valuated was Sample 14 (average of 7.6), which implied a minor range than the other two segments.

Finally, the third cluster (52%) was the most numerous. These consumers gave punctuations lower for all the samples, and only Sample 16, with an average of 6.2, was valuated above 6 (“Like slightly”).

These observations give additional information to the general results discussed earlier. For instance, it seems that while for some consumers (Cluster 1), color of the samples is important, since they are capable of differentiating samples in terms of acceptance, just by looking at them; other consumers (Cluster 2) did not found differences among the beverages when they only evaluated their appearances. Moreover, from this analysis it can be observed that for a group of consumers (Cluster 3), none of the colors of the samples were appreciated.

#### 3.2.2 Internal preference mapping

**Figure 4:**
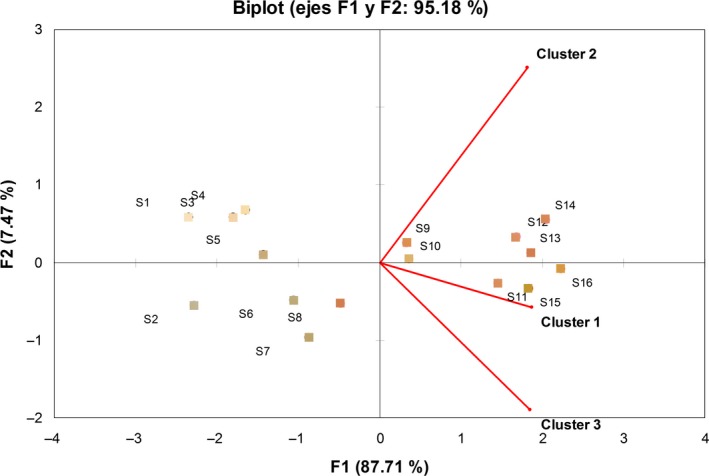
Internal preference mapping defined by the two first preference dimensions from the consumers' cluster

Internal preference mapping refers to the analysis of preference data only, and it was conducted to visualize the general behavior of the clusters of consumers (Figure 4). Two preference dimensions accounted for 95.18% of the total variance, so the third preference dimension was not considered.

All the clusters appear represented in the positive values of the first dimension. Cluster 2 is situated in the higher half of the second dimension, clearly separated from the others clusters and opposite to samples S2, S6, S7, and S8, indicating a relatively lower preference for them (as confirmed in Figure 3). However, clusters 1 and 3 are located in the lower half of the second dimension, opposite to samples S1, S3, S4, and S5, indicating a relatively lower preference for those samples in both clusters. In addition, cluster 1 is much closer to samples 12, 13, and 14, indicating a higher acceptance of those samples as compared with cluster 3 (Figure 3).

Furthermore, samples are divided by the first dimension in two well‐defined groups. In the part of negative values, samples with slightly white and gray appearance are located clearly far from the three clusters. However, all the orangish and reddish samples (with the exception of one sample, S8) appeared in the positive values of the first dimension. This fact showed graphically the consumer tendency to give higher punctuations to the samples with more vivid colors.

## 4 CONCLUSIONS

In this study, color of commercial MFJB was measured by two different techniques confirming that both, SPE and DIA, are appropriate to analyze the appearance of these products. However, consumer study showed that though generally participants significantly preferred samples with orangish appearance instead of those with a whiter look, the low punctuations given to the samples (mean = 5.1) suggested that color of these products was not highly evaluated. This fact should be taken into account by industries as the appearance of food products has a demonstrated influence in food acceptance.

## CONFLICT OF INTEREST

None declared.
